# USE OF PRESCRIPTION MEDICATIONS WITH SOMNOLENCE AS A POTENTIAL ADVERSE EFFECT AMONG OLDER ADULTS IN THE UNITED STATES

**DOI:** 10.1093/geroni/igad104.1561

**Published:** 2023-12-21

**Authors:** Jocelyn Wilder

**Affiliations:** NORC, Chicago, Illinois, United States

## Abstract

Over half of community-dwelling older adults experience sleep disorders, with approximately 40% reporting somnolence or/and excessive daytime sleepiness, associated with an increased risk for cognitive impairment and premature mortality. The use and concurrent use of prescription medication with somnolence as an adverse effect may be an overlooked contributor to this growing problem. This study aims to characterize the use or concurrent use of medications with somnolence as an adverse effect and to assess associations between their use and the prevalence of somnolence (excessive daytime sleepiness or sleep duration of ≥9 hours) using data from the National Social Life, Health, and Aging Project (NSHAP) 2010-2011 (N=3,338). Adjusted prevalence was estimated from multivariable logistic regression models adjusted for socio-demographic measures and the number of medications without somnolence as an adverse effect. The estimated prevalence of somnolence was 54% for those reporting use of 3 or more medications with somnolence as an adverse effect vs. 36.5% for those not using such medications. The use of prescription medications with somnolence as a potential adverse effect was prevalent. Concurrent use of medications with somnolence as an adverse effect was associated with a higher prevalence of somnolence. These findings demonstrate the need for more research to understand the impact of concurrent use of medications with similar adverse side effects on OAs’ health and well-being.

